# The characteristics of the urogenital fascia in the retrorectal space based on male cadaveric dissection and its clinical application

**DOI:** 10.1186/s12893-023-01993-w

**Published:** 2023-04-17

**Authors:** Yi Li, Yan-Bing Ma, Yang Xiao, Guang-Cun Shi, Ya-Min Zhao, Jin-Song Zhou, Cong Tong, Rui-Ting Liu, Li-Kun Yan

**Affiliations:** 1 First Department of General Surgery, Shaanxi Provincial People’s Hospital, Xi’an, Shaanxi 710000 China; 2 Department of Anorectal Surgery, Shaanxi Provincial People’s Hospital, Xi’an, Shaanxi 710000 China; 3 Medical School of Yan’an University, Yan’an, Shaanxi 716000 China; 4 Department of General Surgery, Shandong Provincial Linyi Jinluo Hospital, Linyi, Shandong 276000 China; 5 Department of Human Anatomy, Histology and Embryology, School of Basic Medical Sciences, Xi’an Jiaotong University Health Science Center, Xi’an, Shaanxi 710061 China

**Keywords:** Urogenital fascia, Prehypogastric nerve fascia, Presacral fascia, Parietal pelvic fascia, Retrorectal space, Presacral space

## Abstract

**Background:**

The architecture of retrorectal fasciae is complex, as determined by different anatomical concepts. The aim of this study was to examine the anatomical characteristics of the inferomedial extension of the urogenital fascia (UGF) involving the pelvis to explore its relationship with the adjacent fasciae. Furthermore, we have expounded on the clinical application of UGF.

**Method:**

For our study, we examined 20 adult male pelvic specimens fixed in formalin, including 2 entire pelvic specimens and 18 semipelvic specimens. Our department has performed 466 laparoscopic rectal cancer procedures since January 2020. We reviewed the surgical videos involving UGF preservation and analyzed the anatomy of the UGF.

**Results:**

The bilateral hypogastric nerves ran between the visceral and parietal layers of the UGF. The visceral fascia migrated ventrally at the fourth sacral vertebra, which formed the rectosacral fascia together with the fascia propria of the rectum; the parietal layer continually extended to the pelvic diaphragm, terminating at the levator ani muscle. At the third to fourth sacral vertebra level, the two layers constituted the lateral ligaments.

**Conclusion:**

The double layers of the UGF are vital structures for comprehending the posterior fascia relationship of the rectum. The upper segment between the fascia propria of the rectum and the visceral layer has no evident nerves or blood vessels and is regarded as the " holy plane” for the operation.

## Introduction

Colorectal surgeons must understand the anatomical characteristics of the related fascia surrounding the rectum during rectal cancer surgery. Moreover, anal, urinary, and sexual functions may be preserved if surgery is performed on the correct planes [1–3]. In middle and low rectal cancer surgery, the total mesorectal excision (TME) technique [4] and the pelvic autonomic nerve preservation (PANP) technique [5] are performed on the fasciae of the posterior rectum to preserve the nerves that regulate urogenital and sexual function. These techniques have been heavily debated among colorectal surgeons.

An increasing number of studies have suggested that the fascia covers the hypogastric nerves (HGNs). Researchers have conducted studies on large histological slices and suggested the term “prehypogastric nerve fascia” (pre-HGN fascia) for the fascia overlying the HGN [6]. Similarly, another group described the UGF as a fascia sheath enveloping the ureters and HGNs in the retrorectal space [7]. However, in several studies, the researchers disagreed on the relationship between the retrorectal fascia and the HGNs [3, 8–11]. In another study, by examining 12 formalin-fixed and 12 fresh cadavers, researchers suggested that the urogenital-hypogastric sheath is composed of multiple layers of renal fascia that extend downward to form a sandwich-like compound fascia sheath [12]. These controversial views complicate our understanding of the anatomy of the posterior rectal fascia and the appropriate surgical procedures.

Understanding the anatomy of the UGF has specific clinical guiding significance in colorectal cancer surgery. In our previous anatomy study, we also proposed that the UGF derives from the renal fascia and includes the visceral and parietal layers, [13] namely preperitoneal fascia [14]. Then, in a cadaver study on the efficacy of TME for preserving the UGF, researchers concluded that the true avascular plane is between the fascia propria of the rectum and the UGF [15] Nevertheless, this group did not expound on the idea that the UGF comprises two layers. Although our previous study suggested that the visceral layer of the UGF forms the rectosacral fascia together with the fascia propria of the rectum, [13] an impeccably detailed description of the characteristics of the UGF around the rectum does not exist. In addition, surgical techniques for colorectal cancer that consider the UGF are lacking.

Therefore, we conducted an in-depth study of the anatomy of the UGF in the pelvis and explored its anatomical relationships with surrounding fasciae, nerves, and organs in male formalin-fixed cadavers. We also described the clinical relevance of the UGF while performing mid-low laparoscopic TME.

## Materials and methods

### Cadavers and anatomical dissection

The study was conducted in strict accordance with protocols approved by the Biomedical Ethics Committee of Xi’an Jiaotong University (Ethics Permit Number: 2014 − 0303). The format of the informed consent form was in line with the guidelines of the China Organ Donation Administrative Center. Furthermore, this anatomical study followed the CACTUS guidelines [16]. The inclusion criteria are male adult cadavers, no abdominopelvic injury, deformity, or surgery, and no organ deficiencies. Finally, twenty formalin-fixed male cadavers (45–65 years old) were provided and examined by the Department of Anthropotomy and Histo-Embryology of Xi’an Jiaotong University Health Science Center. And all cadavers were recently formalin-fixed to reduce the bias postmortem changes might cause. All authors underwent rigorous training in this institute before conducting this study.

The pelvis was dissected along the midsagittal plane in 18 cadavers to observe the hemipelvis, and the whole pelvis was examined in the remaining cadavers. The dissections were conducted by two experienced colorectal surgeons from our department utilizing standard surgical instruments (tissue clamps, nontoothed tissue forceps, scalpel, scissors) to avoid complicated visualization techniques.

1. Observing the whole pelvis: The cadavers were cut transversely along the plane of the fourth lumbar vertebra and the perineal plane to observe the entire pelvis.

According to the laparoscopic TME procedure, dissection was performed in the retrorectal space and reached the level of the levator ani muscle. Next, after identifying the peritoneal reflection, Denonvilliers’ fascia was dissected, and its anterior space was observed. Then, dissection extended toward the lateral ligaments with special consideration to maintain the integrity of the fascial continuity.

2. Preparation of the hemipelvis: A hacksaw was used to dissect 18 cadavers along the midline of the sagittal plane from the lower lumbar vertebral spinous process to the coccyx tip to obtain the hemipelvis.

The operation was performed in the sacral promontory between the fascia propria of the rectum and the visceral layer of the UGF. Next, dissection continued between the parietal layer of the UGF and the sacrum.

The main observations are summarized below: (1) the distribution of the UGF in the pelvic cavity and its relationship with surrounding fasciae and (2) the distribution of the UGF with HGNs, ureters, and pelvic plexus.

## Results

**Fig. 1:**
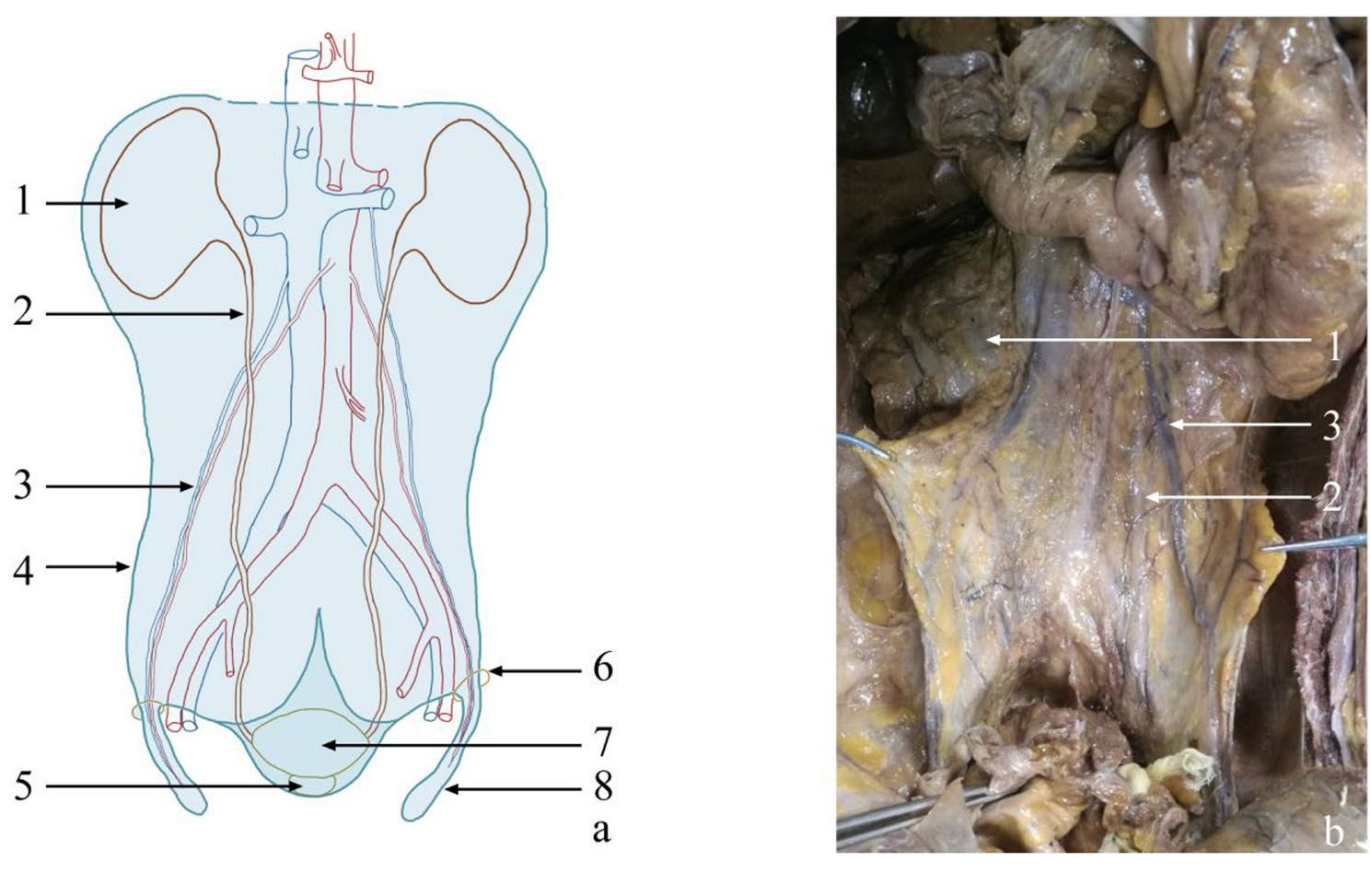
Overall appearance of the UGF [13]. **(a)** Schematic diagram of the anatomical components of the urogenital fascia (UGF). The light-blue shaded portion represents the UGF; the light-blue dotted line indicates the unclear boundary. **(b)** Partial entity of panel **(a)** in a cadaver. (1) kidney; (2) ureter; (3) genital vessel; (4) lateral boundary of the UGF; (5) prostate; (6) deep inguinal ring; (7) bladder; (8) spermatic sheath

**Fig. 2:**
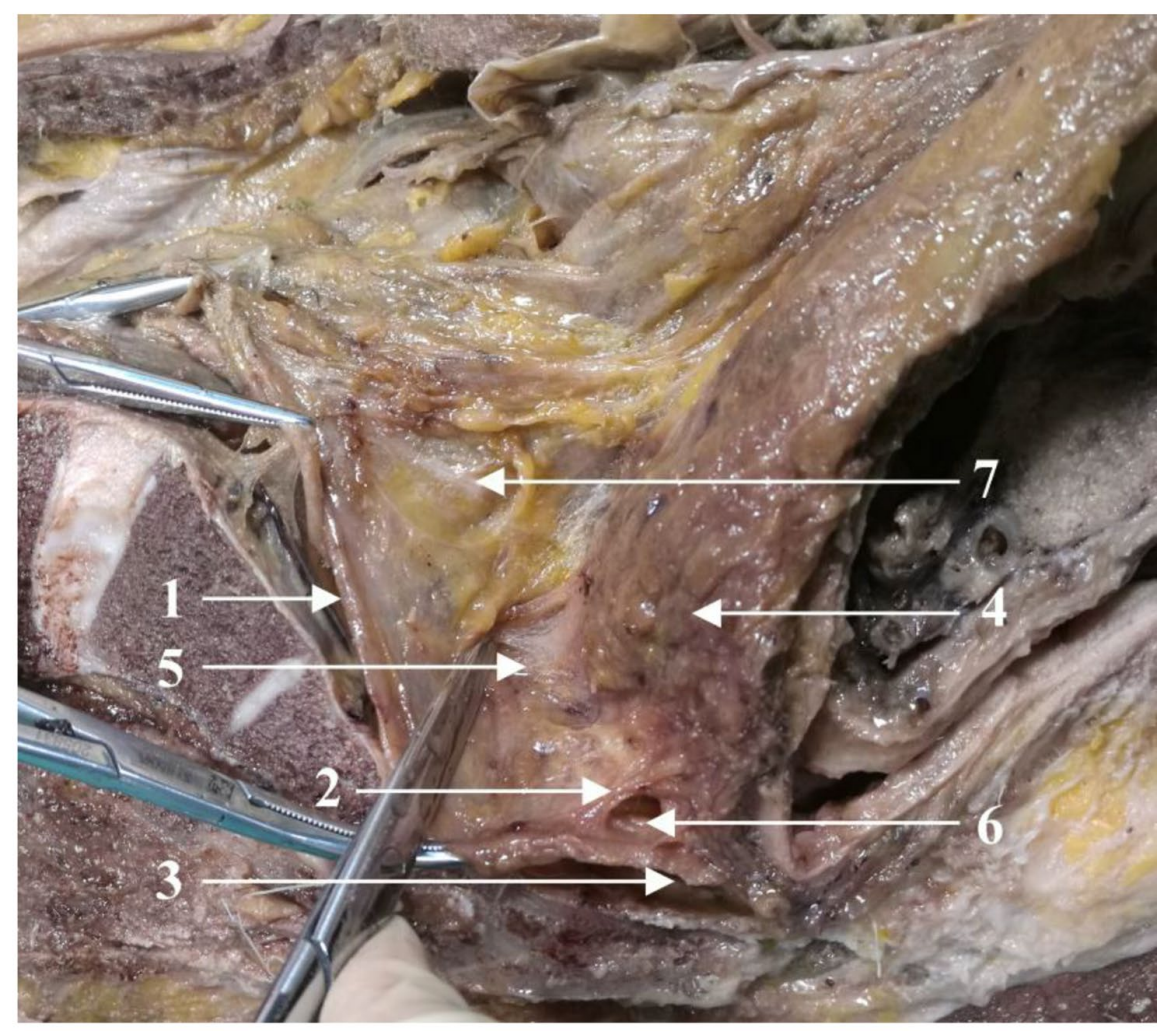
The anatomical characteristics of the UGF posterior to the rectum (sagittal section) (1) urogenital fascia (UGF); (2) rectosacral fascia; (3) the parietal layer of UGF; (4) rectum; (5) upper segment of retrorectal space; (6) lower segment of retrorectal space; (7) left hypogastric nerve

Originating from the renal fascia, the visceral and parietal layers of the UGF extended along the sacrum to the pelvic floor, constituting the presacral fascia. The ureter and the bilateral HGNs ran between the two layers of the UGF (Fig. 1) [13]. The visceral layer was fused with the fascia propria of the rectum at the fourth sacral vertebra to form the rectosacral fascia. However, the parietal layer extended caudally and terminated at the levator ani muscle (Fig. 2).

**Fig. 3:**
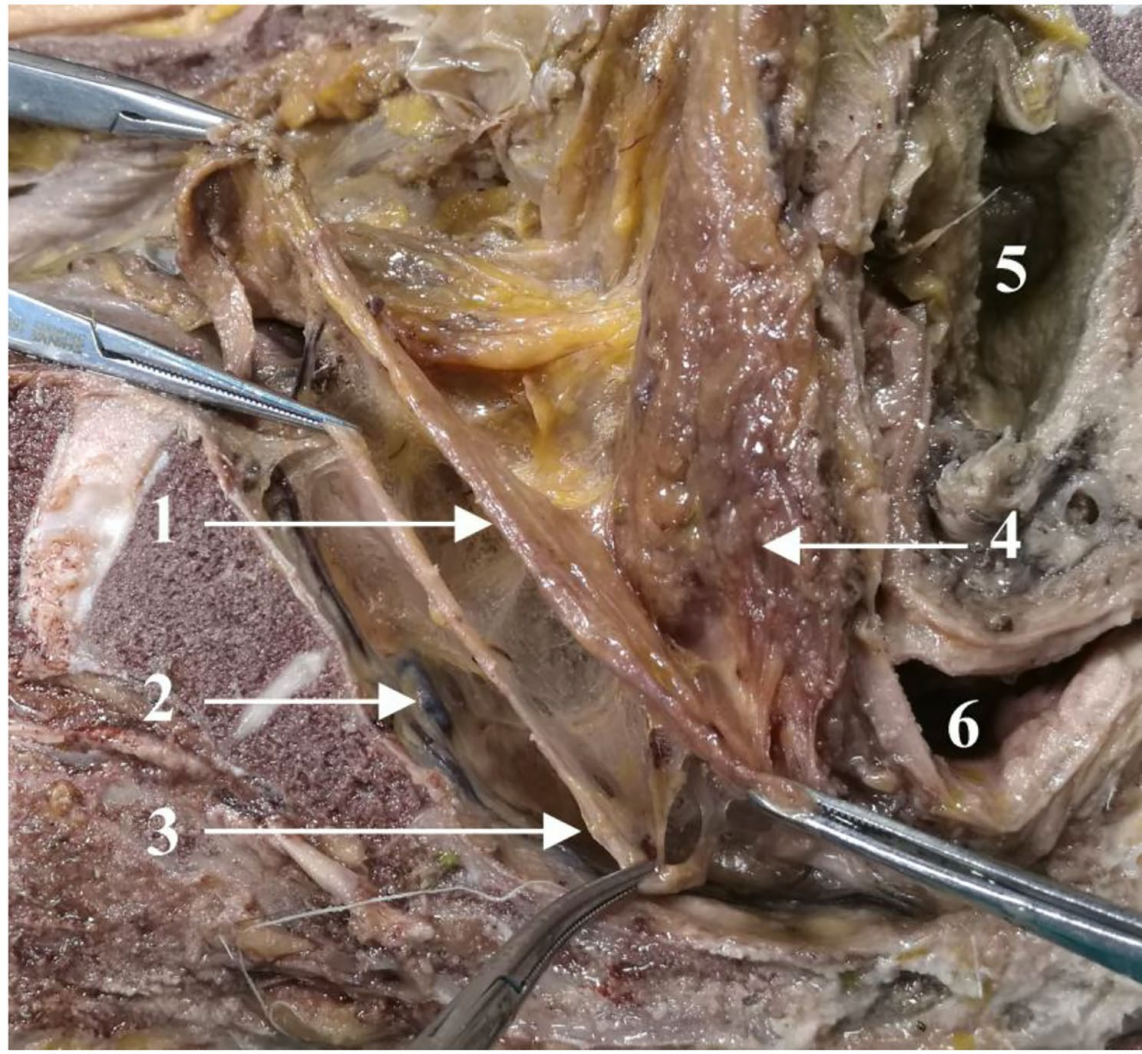
The relationship between the UGF and the presacral fascia (sagittal section) (1) urogenital fascia (UGF); (2) presacral venous; (3) parietal pelvic fascia; (4) fascia propria of the rectum; (5) bladder; (6) rectum

**Fig. 4:**
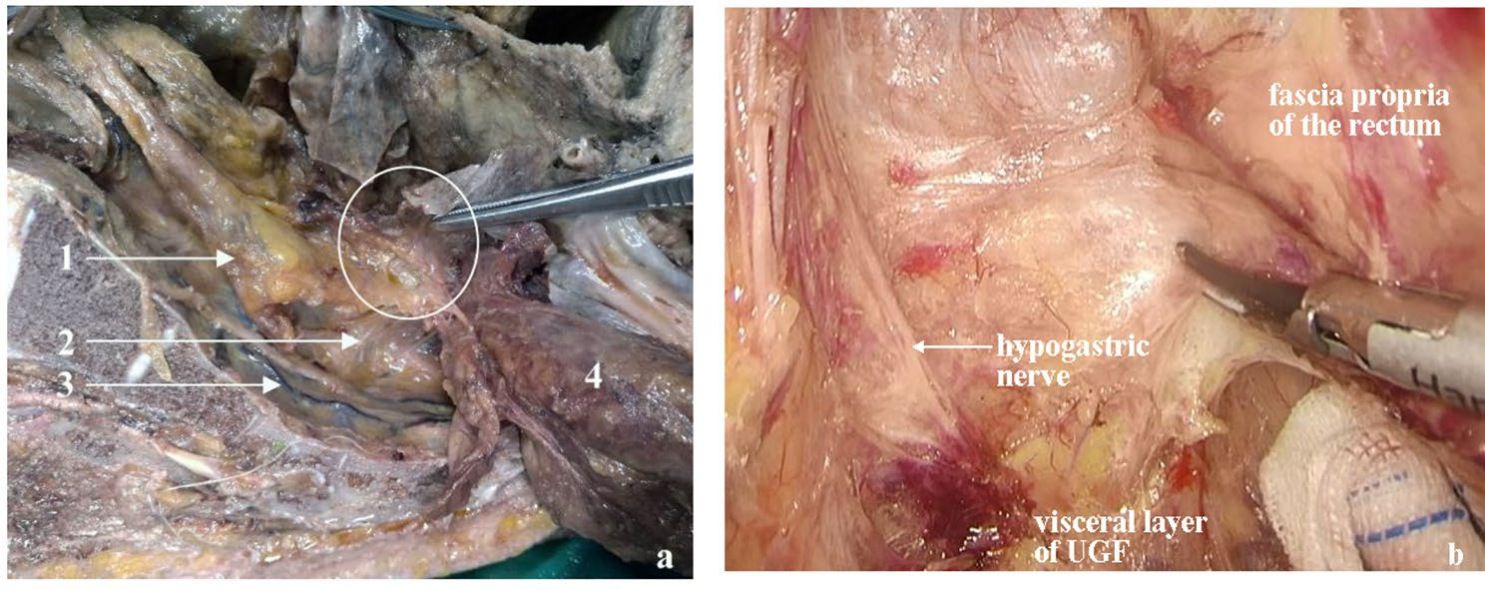
A mesentery-like structure in the lateral wall of the rectum formed by the UGF and the pelvic plexus **(a)** cadaver anatomy; **(b)** laparoscopic observation: (1) urogenital fascia (UGF); (2) pelvic splanchnic nerves; (3) presacral venous; (4) rectum. In panel **(a)**, the parietal pelvic fascia was removed. The white circle represents the attachment of the mesentery-like structure formed by the UGF and the pelvic plexus at the lateral pelvic wall. **(b)** shows that during laparoscopic TME, the separation of the left lateral rectal mesentery (the lateral rectal ligament) is guided by the visceral layer of the UGF to protect the HGN and pelvic splanchnic nerves

Presacral venous were exposed when the parietal pelvic fascia was lifted from the sacrum (Fig. 3). Laterally, the UGF extended along the lateral wall of the pelvis. Furthermore, the pelvic splanchnic nerves originating from the sacral nerves (at the level of the second to fourth sacral vertebra) penetrated the parietal layer of the UGF to join the pelvic plexus. The UGF enveloped the pelvic plexus on the anterior-lateral side, similar to a mesentery structure (Fig. 4).

## Discussion

Our study provides a comprehensive description of the UGF and its features. The UGF, originating from the renal fascia, comprises the visceral and parietal layers and extends downward to the pelvic floor along the sacrum. The UGF envelops the bilateral HGNs and ureters and extends to the pelvis. At the level of the fourth sacral vertebra, the visceral fascia inverts ventrally and forms the rectosacral fascia. However, the parietal layer continues along the sacrum and ends at the levator ani muscle. The parietal pelvic fascia is located on the surface of the sacrum.

The UGF is known to originate from the renal fascia and envelop the ureters and HGNs. However, there are distinct discrepancies in opinions on the composition of the UGF. In a previous study, the ureters and genital vessels were found to be located in the two layers of the UGF, whereas the HGNs were not mentioned [17]. Nevertheless, in another study, the researchers noted that the HGNs and the ureters are located in the two layers of the UGF [7]. In our previous anatomical study, we stated that the double layers of the UGF ensheath the ureters, vas deferens, genital vessels, and superior hypogastric plexus and extend along the sacrum toward the pelvic floor [13]. Notably, the bilateral HGNs and ureters are covered by the visceral layer. Because the UGF envelops the HGNs, it has been termed a “hypogastric sheath” [18]. In another study of 12 formalin-fixed and 12 fresh cadavers, the researchers concluded that the urogenital-hypogastric sheath consists of multiple layers of renal fascia ensheathing the HGNs, ureters, and genital vessels downward to their terminations in the pelvis [12].

Understanding the UGF may be instrumental in comprehensively understanding the terminology used for different anatomical regions located posterior to the rectum, as it comprises visceral and parietal layers. For example, different designations exist for the fascia covering the HGNs [19–21]. These concepts were discussed in a previous study [6]. Because the fasciae in the posterior rectum are complex, it is necessary to avoid challenges in differentiating the fasciae. The UGF refers to the fasciae from a holistic and continuous view, which is beneficial for us to further comprehend the complex terminology used for the fascia. A strength of a previous study involving 21 cadavers was that the researchers demonstrated that the prerenal fascia is continuous with the presacral fascia [11]. Nevertheless, they did not describe it as double-layered. Similarly, in another previous study involving histologic identification, the researchers referred to the fascia covering the HGN as the pre-HGN fascia [6]. Although we did not conduct histological examinations in this study, we utilized the cadavers for careful anatomy and analysis to better illustrate the results. Remarkably, the pre-HGN fascia is the visceral layer of the UGF. The parietal layer of the UGF may be referred to as the post-HGN fascia, as it is dorsal to the HGNs and extends along the sacrum. Therefore, the presacral fascia and the pre-HGN fascia are part of the UGF. The double layers of the UGF enveloping the HGNs are the basis for differentiating related fasciae.

In other studies, researchers have shown multiple layers of fasciae in the posterior rectum. Based on macroscopic dissection and histology, a previous study detailed that the parietal pelvic fascia comprises 2 lamellae ensheathing the autonomic pelvic nerves [22]. Notably, we refer to the parietal pelvic fascia here as the UGF. However, the inner lamellae that are inverted ventrally to the rectum and form the rectosacral fascia have not been described. The UGF is more detailed, with the visceral layer of the UGF reversing ventrally to form the rectosacral fascia at the fourth sacral vertebra. This description was consistent with a previous description [22]. In a combined retrospective and prospective study, researchers reported that the rectosacral fascia terminates at the pre-HGN fascia at the level of the fourth sacral vertebra and comprises two layers (upper and lower) [23]. Based on our results, we concluded that the upper and lower segments of the rectosacral fascia they referred to are the visceral and parietal layers of the UGF. Notably, we used the parietal pelvic fascia instead of the piriformis fascia [11] and the presacral fascia [22] referred to in other studies, as they were both a continuation of the transversalis fascia into the pelvis [12]. By combining our results with those of other studies, we concluded that the fasciae between the rectum and the sacrum are the fascia propria of the rectum, the double layers of the UGF, and the parietal pelvic fascia. This conclusion may eliminate confusion regarding fascial terminology to help us better understand key anatomical features.

The mesentery-like structures formed by the UGF and the pelvic plexus in the lateral wall help us to comprehend the lateral rectal ligament (Fig. 4). Previous studies presented different views on the lateral rectal ligament [3, 9, 24, 25]. Similar to our study, another study also observed fascia covering branches of the pelvic plexus, which anchor the rectum to the lateral pelvic wall [12]. In addition, by analyzing and discussing rectal cancer cases, a previous study stated that the lateral ligament is a pathway of blood vessels and nerve fibers extending toward the rectum and lymphatic vessels from the lower rectum toward the iliac lymph nodes [9]. Therefore, instead of the lateral rectal ligament, we propose the symmetrical lateral rectal mesentery to better understand the lateral rectal nerves, blood vessels, and lymphatic metastases. During the laparoscopic TME, the separation of the lateral rectal mesentery (the lateral rectal ligament) is guided by the visceral layer of UGF to protect the HGNs and pelvic splanchnic nerves, as shown in Fig. 4 (b).

**Fig. 5:**
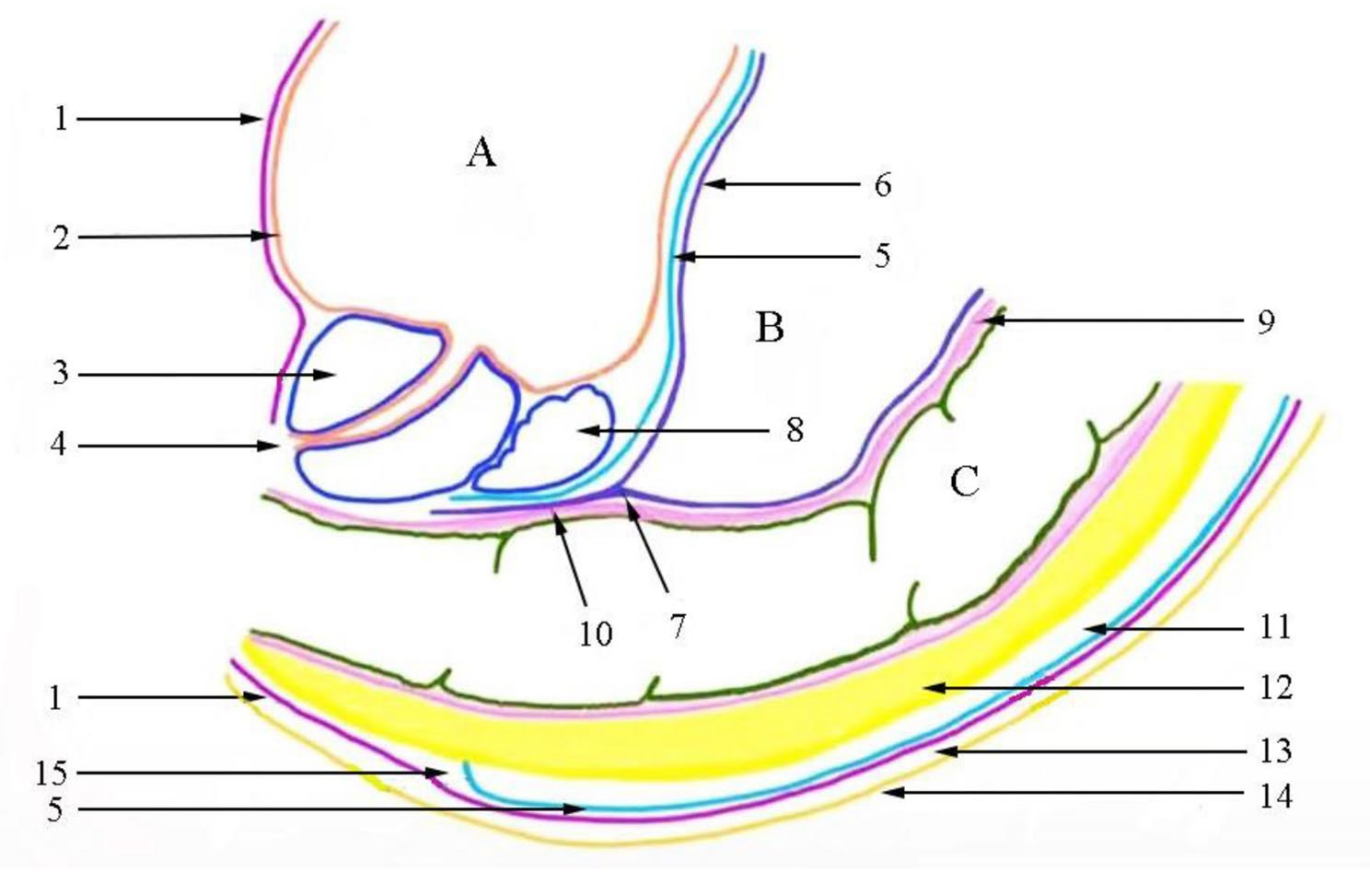
The relationship between the UGF and the perirectal fascia (sagittal section) (1) parietal layer of urogenital fascia (UGF); (2) bladder fascia; (3) prostate; (4) urethral orifice; (5) visceral layer of UGF; (6) peritoneum; (7) peritoneal reflection; (8) seminal vesicle; (9) rectal muscular layer; (10) Denonvilliers’ fascia; 11. upper segment of retrorectal space; 12. mesorectum; 13. presacral space; 14. parietal pelvic fascia; 15. lower segment of retrorectal space; **(A)** bladder; **(B)** abdominal cavity; **(C)** rectum

**Fig. 6:**
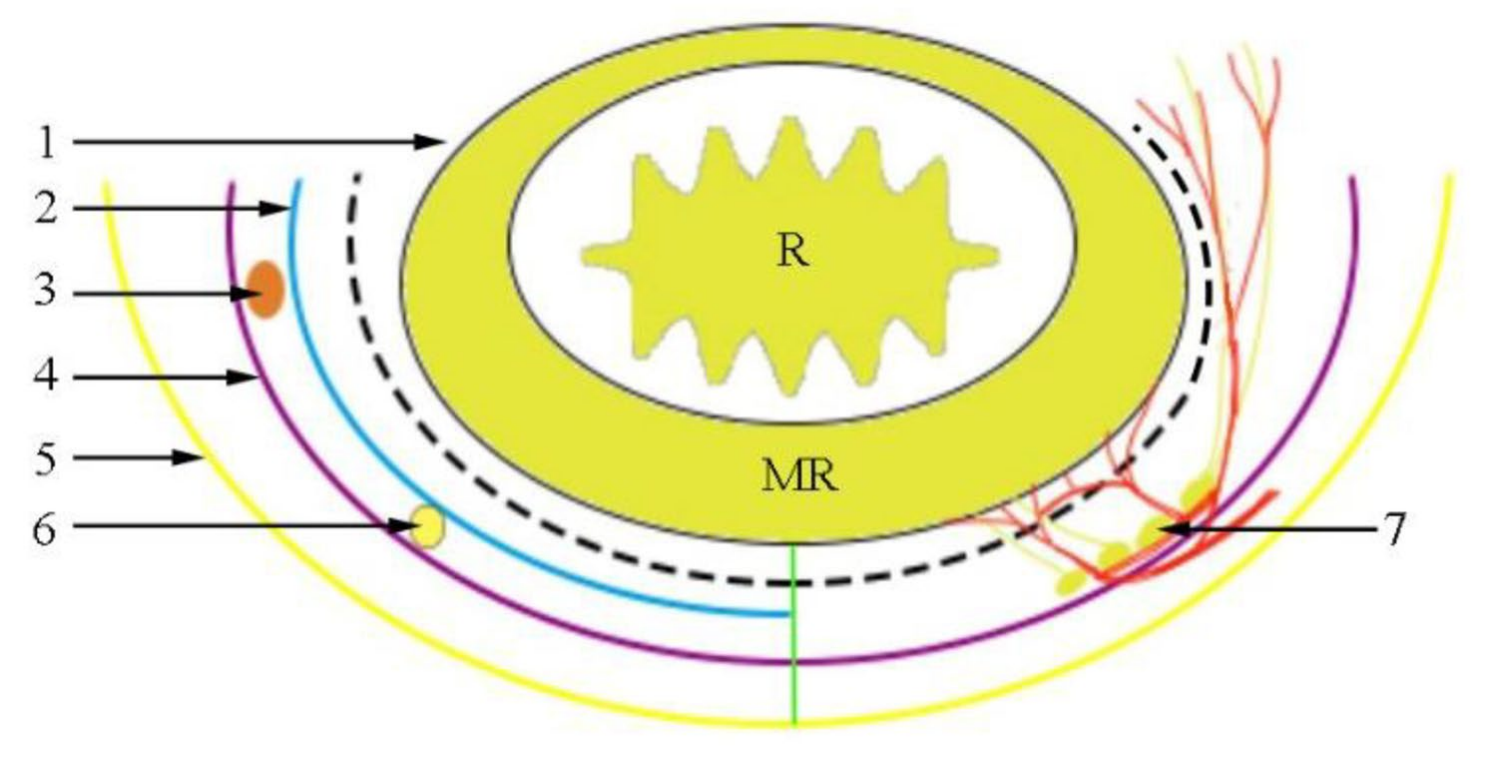
Schematic diagram of the perirectal fascia from the cross-section. 1. Fascia propria of the rectum; 2. visceral layer of urogenital fascia (UGF); 3. ureter; 4. parietal layer of UGF; 5. parietal pelvic fascia; 6. hypogastric nerve; 7. pelvic plexus; R: rectum; MR: mesorectum. The dotted line refers to the surgical operation plane

According to the UGF’s characteristics, we propose that the retrorectal space is divided into upper and lower segments by the visceral layer of the UGF. On the cranial side of the fourth sacral vertebra, the space between the fascia propria of the rectum and the visceral layer may be called the upper segment of the retrorectal space. Correspondingly, the lower segment of the retrorectal space lies between the visceral and parietal layers on the caudal side of the fourth sacral vertebra. The presacral space is located between the parietal layer of the UGF and the piriformis fascia [22]. Based on this, our team drew a sagittal pattern of the UGF posterior to the rectum (Fig. 5). No nerves or blood vessels are present in the upper segment of the retrorectal space, and the lower segment contains branches of the pelvic plexus (Fig. 6). The parietal layer extends toward the pelvic floor, possibly terminating in the levator ani muscle, which may need to be further confirmed by cadaver studies and in clinical practice.

**Fig. 7:**
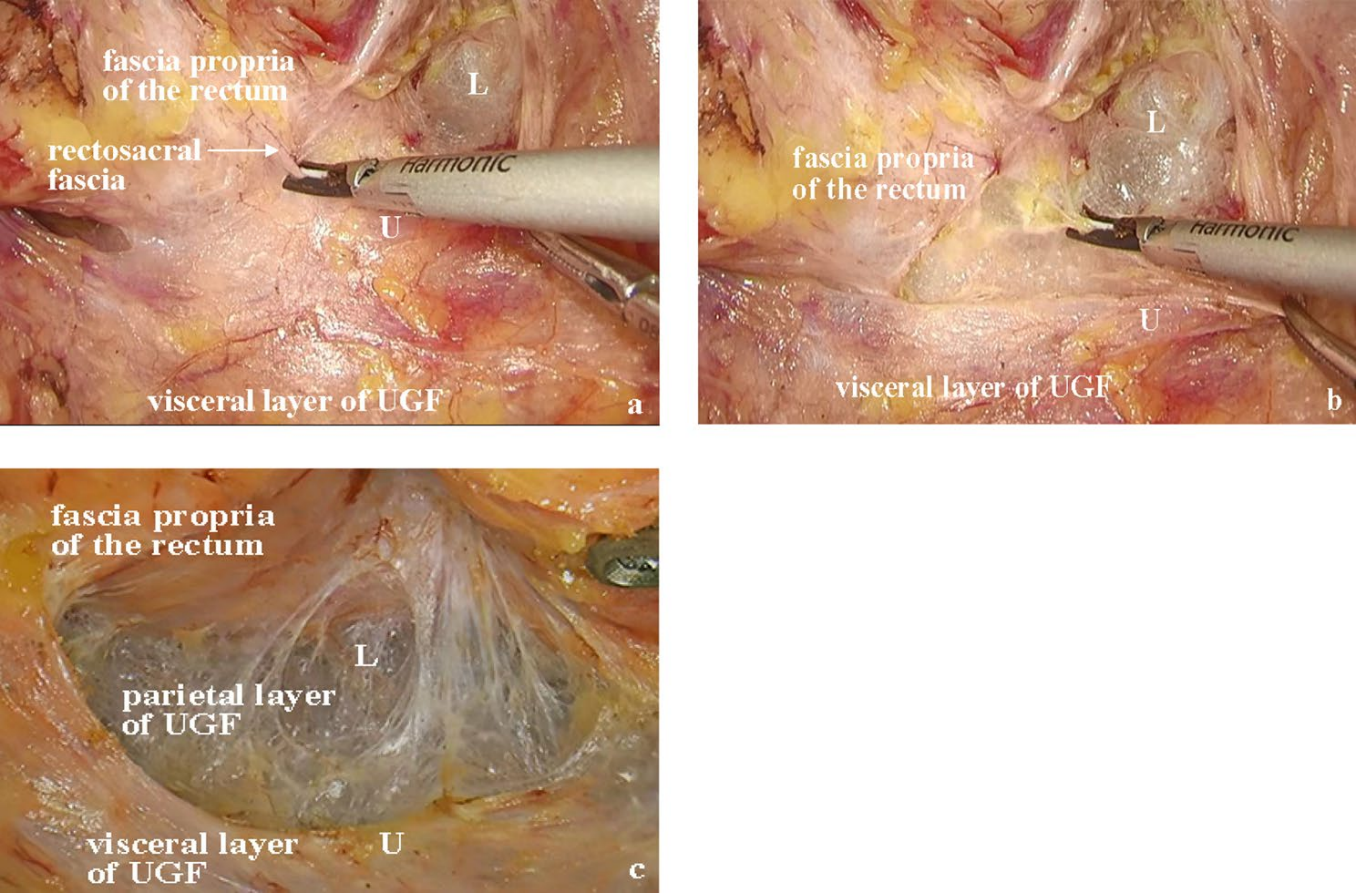
Retrorectal space during laparoscopic TME based on UGF **(a)** The plane between the fascia propria of the rectum and the parietal layer of urogenital fascia (UGF) is the true “holy plane” for laparoscopic total mesorectal technique (TME) dissection. **(b)** When cutting the rectosacral fascia, the operation is between the low segment of the retrorectal space. **(c)** The operation is conducted in the lower segment of retrorectal space. U, upper segment of retrorectal space; L, lower segment of retrorectal space

Based on the above perspectives, the optimal operation plane of the retrorectal space is the upper segment of the retrorectal space between the fascia propria of the rectum and the visceral layer. This plane corresponds to the “holy plane” of TME. Notably, after the operation reaches or crosses the rectosacral fascia, separation is performed in the lower segment of the retrorectal space (Fig. 7). However, the surgical plane always lies between the two layers of the UGF, inevitably causing damage to the related nerves. Dissection separates the parietal layer of the UGF and allows entry into the presacral space. As a result, the sacral nerves and presacral venous may be exposed, and an inappropriate operation leads to bleeding.

This study is the first to propose the double layers UGF as a theoretical guide in TME and expound on the relevant noteworthy details. Comprehending the characteristics of UGF and discerning the relevant, distinct fasciae are fundamental for colorectal surgeons to distinguish the surgical-related plane better, avoid nerve injury or bleeding, and conduct TME faster at the criteria plane. Establishing the retrorectal space based on the characteristics of UGF also provides a guide for separating the lateral rectal mesentery (namely, lateral rectal ligament) and discerning Denonvilliers’ fascia adjacent to the UGF [26]. Moreover, a complete comprehension of the distribution characteristics of the two-layered structure of UGF enhances the understanding of the lymphatic metastasis pattern of the low rectum and the pathway of retrorectal space infection spreading to the cranial side.

## Limitations

This study has two limitations. The study of formalin-fixed cadavers instead of fresh cadavers is an inherent limitation, as findings could be ascribed to postmortem degenerative changes. In addition, we did not perform histological examinations in the present study. Such examinations will be conducted in future anatomical studies.

## Conclusion

The double layers of the UGF are vital structures for understanding the posterior fascial relationship of the rectum. The parietal and visceral layers of the UGF divide the retrorectal space into upper and lower segments. Furthermore, the upper segment between the fascia propria of the rectum and the visceral layer has no evident nerves or blood vessels and is the “holy plane” for conducting the operation.

## Data Availability

The datasets generated and analyzed during the current study are available from the corresponding author on reasonable request.
